# Editorial on the Special Issue “Advances in Nanogels”

**DOI:** 10.3390/gels8120835

**Published:** 2022-12-17

**Authors:** Chien-Chi Lin, Emanuele Mauri, Filippo Rossi

**Affiliations:** 1 Department of Biomedical Engineering, Indiana University-Purdue University Indianapolis, Indianapolis, IN 46202, USA; 2 Department of Engineering, Università Campus Bio-Medico di Roma, Via Álvaro del Portillo 21, 00128 Rome, Italy; 3 Department of Chemistry, Materials and Chemical Engineering “G. Natta”, Politecnico di Milano, Via Mancinelli 7, 20131 Milan, Italy

In recent decades, the rise of nanotechnology has led to the design of innovative nano-biomaterials which are used to improve pharmacological therapies and assist with disease diagnosis. Among them, nanogels represent one of the most promising nanocarriers for tunable drug release and selective cell targeting. In general, nanogels are composed of physically/chemically cross-linked polymer chains organized in a three-dimensional nanostructure. Like their bulk gel counterpart, nanogels are capable of imbibing a large quantity of water and undergoing reversible swelling/deswelling. This enhances the dispersion stability, the interactions with physiological compartments and the bioavailability of the loaded drugs or proteins. Other unique properties include highly tunable porosity, hydrophilicity, stability, size, and charge by means of modulating chemical compositions of the polymers or grafting additional functionalities on the nanogel surface. In particular, functionalization with specific chemical linkages or molecules promotes controlled release of the payload according to specific external stimuli (e.g., redox agents, enzymatic activities, pH and temperature variations), extending the curative benefits over time. At the same time, the configuration of core–shell nanogels or the development of coating layers are two potential alternatives to modulate the nanosystem–cell interactions. Altering these properties affects cellular uptake and provides a means for selective cell internalization, which represents a major challenge for the clinical administration of nanosystems.

Starting with the contributions of Prof. Akiyoshi (1993) and Prof. Vinogradov (1999) that designed, respectively, the first physical and chemical nanogels, many innovative proposals have been studied in recent years, combining in vitro and in vivo applications in a range of medical fields [1,2].

Nanogels can be prepared by natural polymers, such as hyaluronic acid (HA) and chitosan. Malytskyi and coworkers [3] obtained stable nanogels from HA and chitosan using tripolyphosphate as a cross-linker, taking advantage of the ionic gelation mechanism. The nanogels were highly stable even in presence of enzymes, which is advantageous in biomedical applications. Chitosan was also used by Saraogi and coworkers [4] to produce nanogels using ionization methods that can then be loaded with pravastatin for hyperlipidemia treatment. The sustained release, together with the compatibility with excipients and high hemolytic activity, support the prospective use of orally administered pravastatin-loaded nanogel as an effective and safe nano-delivery system in hyperlipidemia treatment.

Nanogels are commonly prepared by synthetic polymers. Md and coworkers [5] used an emulsion–diffusion–evaporation technique with a three-level, three-factor Box–Behnken design to produce poly (D, L-lactic-co-glycolic acid)-based nanogels. The developed formulations showed excellent flux across the skin layer in the skin permeation study, and skin probe fluorescent dye in confocal microscopy revealed significant penetration of NPs into the skin, suggesting their possible use in skin-cancer treatment. Functionalization strategies can then be added to preformed nanogels for specific and selective cancer-cell targeting. Campora and coworkers [6] decorated poly(N-isopropylacrylamide) colloids with acrylic acid and coupled them with folic acid, targeting the folate receptors overexpressed by cancer cells and the chemotherapeutic drug doxorubicin. This approach supports their possible use in selective cell-cancer treatment, with consequent amelioration of chemotherapeutic strategies.

The high versatility on nanogels is also described in the four review papers in this Special Issue: Mauri et al. [7] proposed an interesting focus on the recent techniques used in nanogel design, highlighting key upgrades in terms of methodologies, microfluidics, and 3D printing. Macromolecules and biomolecules can indeed be combined to create ad hoc nanonetworks according to the final curative goals, maintaining the criteria of biocompatibility and biodegradability. From a materials perspective, as described by Kaewruethai and coworkers [8], nanogels were designed as core–shell structures that demonstrated efficient responsiveness to different stimuli such as temperature, pH, reductive environment, or radiation. Stawicki et al. [9] focused on the use of nanogels as vehicles to cross the blood–brain barrier, providing local and systemic drug-delivery systems in the treatment of brain cancer. Indeed, the current ongoing development allows patient-centered treatment that can be considered a promising tool for the management of brain cancer. Moreover, other targets can be addressed, such as wound healing, in which the ability of nanogels to carry and release drugs is extremely promising, as discussed by Qadir and coworkers [10]. 

In summary, the present Special Issue provides an overview of the different potential approaches to synthetizing smart nanogels for controlled drug delivery, with particular emphasis on the functionalization strategies of these nanocarriers and their application in in vitro and/or in vivo models. We hope that this Special Issue will offer good insight into recent studies on nanogels and their applications. We thank all the research teams that contributed to “Advances in Nanogels”. 

## References

[1] Vinogradov S., Batrakova E., Kabanov A. (1999). Poly(ethylene glycol)-polyethyleneimine NanoGel (TM) particles: Novel drug delivery systems for antisense oligonucleotides. Colloids Surf. B Biointerfaces.

[2] Pinelli F., Perale G., Rossi F. (2020). Coating and Functionalization Strategies for Nanogels and Nanoparticles for Selective Drug Delivery. Gels.

[3] Malytskyi V., Moreau J., Callewaert M., Henoumont C., Cadiou C., Feuillie C., Laurent S., Molinari M., Chuburu F. (2022). Synthesis and Characterization of Conjugated Hyaluronic Acids. Application to Stability Studies of Chitosan-Hyaluronic Acid Nanogels Based on Fluorescence Resonance Energy Transfer. Gels.

[4] Saraogi G.K., Tholiya S., Mishra Y., Mishra V., Albutti A., Nayak P., Tambuwala M.M. (2022). Formulation Development and Evaluation of Pravastatin-Loaded Nanogel for Hyperlipidemia Management. Gels.

[5] Md S., Alhakamy N.A., Neamatallah T., Alshehri S., Mujtaba M.A., Riadi Y., Radhakrishnan A.K., Khalilullah H., Gupta M., Akhter M.H. (2021). Development, Characterization, and Evaluation of α-Mangostin-Loaded Polymeric Nanoparticle Gel for Topical Therapy in Skin Cancer. Gels.

[6] Campora S., Mohsen R., Passaro D., Samir H., Ashraf H., Al-Mofty S.E.-D., Diab A.A., El-Sherbiny I.M., Snowden M.J., Ghersi G. (2021). Functionalized Poly(N-isopropylacrylamide)-Based Microgels in Tumor Targeting and Drug Delivery. Gels.

[7] Mauri E., Giannitelli S.M., Trombetta M., Rainer A. (2021). Synthesis of Nanogels: Current Trends and Future Outlook. Gels.

[8] Kaewruethai T., Laomeephol C., Pan Y., Luckanagul J.A. (2021). Multifunctional Polymeric Nanogels for Biomedical Applications. Gels.

[9] Stawicki B., Schacher T., Cho H. (2021). Nanogels as a Versatile Drug Delivery System for Brain Cancer. Gels.

[10] Qadir A., Jahan S., Aqil M., Warsi M.H., Alhakamy N.A., Alfaleh M.A., Khan N., Ali A. (2021). Phytochemical-Based Nano-Pharmacotherapeutics for Management of Burn Wound Healing. Gels.

